# Performance of the hematology analyzer XN-31 prototype in the detection of Plasmodium infections in an endemic region of Colombia

**DOI:** 10.1038/s41598-021-84594-y

**Published:** 2021-03-04

**Authors:** Lina Zuluaga-Idárraga, Alexandra Rios, Verónica Sierra-Cifuentes, Edwar Garzón, Alberto Tobón-Castaño, Ikki Takehara, Yuji Toya, Munehisa Izuka, Kinya Uchihashi, Tatiana M. Lopera-Mesa

**Affiliations:** 1 Grupo Malaria, Facultad de Medicina, Universidad de Antioquia, Carrera 53 No. 61–30, Lab 610, Medellín, Colombia; 2 Sysmex Corporation, 1-5-1 Wakinohama-Kaigandori, Chuo-ku, Kobe, Hyogo 651-0073 Japan

**Keywords:** Biological techniques, Microbiology, Diseases, Medical research

## Abstract

Early and accurate diagnosis is critical in reducing the morbidity and mortality associated with malaria. Microscopy (MI) is the current diagnostic gold standard in the field; however, it requires expert personnel, is time-consuming, and has limited sensitivity. Although rapid diagnostic tests for antigen detection (RDTs) are an alternative to diagnosis, they also have limited sensitivity and produce false positive results in detecting recent past infection. The automated hematology analyzer XN-31 prototype (XN-31p) (Sysmex Corporation, Kobe, Japan) is able to identify plasmodium-infected erythrocytes, count parasitemia and perform complete blood-cell counts within one minute. The performance of the XN-31p in diagnosing malaria was evaluated and compared with real-time polymerase chain reaction (qPCR), MI and RDT in an endemic area of Colombia where *Plasmodium falciparum* and *Plasmodium vivax* are present. Acute febrile patients were enrolled from July 2018 to April 2019 in Quibdó, Colombia. Malaria diagnoses were obtained from MI and RDT in the field and later confirmed by qPCR. Venous blood samples in EDTA were processed with an XN-31p in the field. Sensitivity, specificity, positive/negative predictive values, and the likelihood ratios of positive and negative tests were calculated with respect to the results from qPCR, MI and RDT. The intraclass correlation coefficient (ICC) and Bland–Altman plot were used to evaluate the concordance in the parasitemia with respect to MI. A total of 1,754 subjects were enrolled. The mean age was 27.0 years (IQR 14–44); 89.6% were Afro-Colombians, 94.3% lived in urban areas and 0.91% were pregnant. With respect to qPCR, the XN-31p showed a sensitivity of 90% (95% CI 87.24–92.34) and a specificity of 99.83% (95% CI 99.38–99.98) in detecting *Plasmodium* spp.; both parameters were equivalent to those for MI and RDT. Using MI as the reference, the XN-31p showed a sensitivity of 98.09% (95% CI 96.51–99.08), a specificity of 99.83% (95% CI 99.4–99.98), an ICC of 0.85 (95% CI 0.83–0.87) and an average difference of − 3096 parasites/µL when compared with thick-smear MI and an ICC of 0.98 (95% CI 0.97–0.98) and an average difference of − 0.0013% when compared with thin-smear MI. The XN-31p offers a rapid and accurate alternative method for diagnosing malaria in clinical laboratories in areas where *P. falciparum* and *P. vivax* cocirculate.

## Introduction

Malaria infection remains a significant public health problem worldwide. In 2018, an estimated 228 million cases of malaria infection and 405,000 associated deaths were reported. Africa has the largest burden of malaria morbidity (93%), followed by South-East Asia (3.4%) and the Eastern Mediterranean region (2.1%). Five species of the genus Plasmodium cause human infection: *Plasmodium falciparum, Plasmodium vivax, Plasmodium ovale*, *Plasmodium malariae* and the zoonotic parasite *Plasmodium knowlesi.* The most prevalent and fatal species worldwide is *P. falciparum*, accounting for 99.7% of estimated malaria cases in Africa. Globally, *P. vivax* causes 3.3% of estimated cases, with 53% of the *P. vivax* burden being in the South-East Asia region. *P. vivax* is the predominant parasite in the Americas, causing 75% of malaria cases^1^.

Although there has been a decrease in malaria morbidity and mortality rates since 2010, significant efforts are needed to approach the WHO global targets by 2030^2^. Early and accurate diagnosis is one of the most important strategies for malaria control and elimination; therefore, the WHO recommends that all patients with suspected malaria have their diagnoses confirmed by microscopy (MI) or a rapid diagnostic test (RDT) before treatment^2^.

Malaria diagnosis is performed routinely by morphologic identification of the parasite through MI, which is considered the gold standard test for diagnosis in clinical settings. This diagnostic method is low cost, allows quantification of parasitemia, is useful during follow-up of parasitemia clearance and allows the possible identification of other pathogens. However, MI is time consuming, requests trained personal and has an operator-dependent limit of detection, as expert microscopists can identify up to 10–50 parasites/μL, while nonexperts have detection levels in the range of 100–500 parasites/μL^3^.

Alternatives to MI include rapid diagnostic tests (RDTs), which are point-of-care tests that can be performed quickly, are easy to use and do not require trained personnel. Although RDTs have improved access to malaria diagnoses outside health facilities, they have important limitations, such as the inability to quantify parasitemia and differentiate parasite stages and a high limit of detection (100–200 parasites/μL)^3,4^. In addition, HRP2-based RDTs are not useful for following up patients on treatment, since this antigen can circulate for weeks after parasite clearance^5^.

Molecular methods such as nucleic acid amplification techniques (NAATs), including polymerase chain reaction (PCR), provide superior sensitivity over other methods, with reported detection thresholds < 5 parasites/μL and the highest specificity^6–8^. Parasite quantification is possible, but it is not available in clinical setting. They present disadvantages for implementation in the field since they are expensive and time-consuming, require trained personnel, yield results that have little use during follow-up after treatment, and do not allow differentiation of parasite stages. However, PCR is very useful in reference laboratories^3^.

The search for alternative diagnostic methods for the detection and quantification of Plasmodium infections is ongoing. Automated hematology analyzers can offer fast, sensitive and cost-effective assessments of all suspected malaria infections, as they reduce analytical time and improve accuracy^9–11^. The automated hematology analyzer XN-31 prototype (XN-31p) (Sysmex, Japan) is a novel analyzer model that has been optimized to detect Plasmodium-infected red blood cells (iRBCs) circulating in human blood using a combination of optical fluorescence techniques, flow cytometry and laser-optical recognition by a violet semiconductor 405 nm laser beam. In this study, an XN-31p was used that can perform a complete blood count (CBC) and classify different blood cell populations, as well as identify iRBCs and count parasitemia within one minute with a limit of quantification of 20 iRBCs/μL^10^.

While the XN-31p was designed for in vitro diagnosis use, the XN-30, which had been developed for research use, had been previously evaluated in several studies. Both analyzers have the same physical characteristics, hardware configuration, and reagent composition^12^.

Tougan and colleagues reported the use of the XN-30 in recognizing *P. falciparum* parasites cultured in vitro, which permitted the identification of parasite stages and the sensitive and reproducible calculation of parasitemia^13^. Later, the XN-30 was applied in the screening new antiplasmodial compounds^14^. In addition, the XN-30 system permitted the simultaneous measurement of iRBCs in mouse blood samples infected with rodent malarial parasites and hematological evaluation^15^.

Two previous studies evaluated the performance of the XN-30 in a clinical setting^10,11^. Pillay and colleagues validated the parameters of the XN-30, such as the limit of blank (LoB), limit of detection (LoD), limit of quantitation (LoQ), carryover, precision and stability; in addition, they used 191 samples with malaria diagnoses previously confirmed by MI and RDT to assess the identification of iRBCs by the XN-30^11^. Post and colleagues evaluated the performance of the XN-30 in diagnosing malaria and compared it to the performance of MI and qPCR in 908 clinical samples from an endemic region of Burkina Faso, which is hyperendemic for *P. falciparum;* the sensitivity and specificity of XN-30 were, respectively, 98.7% and 99.4% relative to MI and 70.9% and 99.6% relative to qPCR^10^. Previous studies have shown the potential application of the XN-30 for malaria diagnosis in the clinical laboratories of endemic regions; however, no evaluation has been performed for the identification of Plasmodium species by the XN-30 in a clinical setting with cocirculating *P. falciparum* and *P. vivax*. In addition, the concordance in the parasite count with respect to MI has not been evaluated.

The primary aim of this study was to evaluate the performance of the XN-31p in diagnosing malaria using venous blood and compare it with that of qPCR, MI and RDT in an endemic region of Colombia. Additionally, a pilot study was performed to evaluate the concordance in the qualitative and quantitative detection of Plasmodium infection between the XN-31p with finger-prick blood and with venous blood.

## Methods

### Study design

An observational cross-sectional study of diagnostic accuracy was carried out to evaluate the performance of the XN-31p in the detection of Plasmodium infections in a clinical setting. Sociodemographic and clinical data were collected from each subject before conducting the diagnostic test (XN-31p, MI, RDT). Samples were tested by the XN-31p, RDT and MI simultaneously at a field site, and a reference standard test (qPCR) was performed later in the reference laboratory at the University of Antioquia, Medellin city. The Standards for Reporting Diagnostic Accuracy Studies (STARD) guidelines were used in this study^16^.

### Study site

The study was conducted in the municipality of Quibdó, Chocó, Colombia (5.6956° N, 76.6498° W), which is located in the Pacific coast region (Supplementary Fig. 1). Malaria transmission is present in the rural and periurban settings in Chocó, and the main economic activity is gold, silver, and platinum mining. Chocó accounted for 27.3% of the total malaria cases in Colombia in 2018, with an annual parasite index (API) (number of cases/1,000 habitants) of 31.6 (high risk); uncomplicated malaria cases were attributed mainly to *P. falciparum* (57.5%), *P. vivax* (38.3%) and mixed infection (*P. falciparum/P. vivax)* (4.1%)^17^. Quibdó is the capital of Chocó state, and it reported 6.7% of all malaria cases in Colombia in 2018 from periurban areas^18^. The participants of this study were enrolled at the Local Hospital Ismael Roldán Valencia (HLIRV), which is the reference hospital for patients with suspected uncomplicated malaria from Quibdó and other nearby municipalities.

### Sample size estimation

The sample size was estimated assuming an expected sensitivity and specificity for the XN-31p of 80% and 90%, respectively, relative to qPCR (gold standard test) for the diagnosis of malaria. The sample size was calculated assuming an expected malaria prevalence of 15% in patients from Quibdó with acute febrile illness, with a 95% confidence interval and 5% error, corresponding to 1,640 participants. A pilot study for evaluating the performance of the XN-31p with finger-prick samples included a convenience sample of 60 participants, 30 with Plasmodium infection (*P. falciparum* n = 15 and *P. vivax* n = 15) and 30 uninfected, according to the MI diagnosis made in the field. These subjects were selected among those enrolled in the framework for assessing the performance of the XN-31p using venous blood.

### Participants

Participants with acute febrile syndrome were enrolled according to the following eligibility criteria: > 2 years old, body weight > 12 kg, fever at enrollment (axillary temperature > 37.5 °C) or history of fever 72 h prior to admission, less than 7 days of fever evolution, no other apparent localized cause of fever, and residence in any malaria transmission area. The participants were enrolled consecutively as they were admitted to the emergency and outpatient services of the HLIRV from July 2018 to April 2019.

### Data and sample collection

All eligible participants were invited to participate and signed informed consent forms. At enrollment, a clinical evaluation was performed by a physician; information including demographic characteristics, current disease, history of malaria episodes, and use of preventive measures for malaria was recorded. A finger prick blood sample (20 µL) was obtained to determine the malaria diagnosis by MI. Venous blood samples (4 mL) were collected from each subject in EDTA tubes and analyzed by the XN-31p; the same sample was used for the malaria RDT test and analyzed by an XN-450 Sysmex analyzer, which had been approved for clinical use, to obtain subject hematological parameters. An aliquot of this sample (500 µL) was frozen in liquid nitrogen and sent to the reference laboratory at University of Antioquia in Medellín for later confirmation of the malaria diagnosis by qPCR (within the next month). When a participant was selected for the pilot study using finger-prick blood, an additional capillary sample (100 µL) was obtained using a high-blood volume lancet and microtubes containing EDTA.

### Diagnostic test methods

#### XN-31 prototype

This study included an XN-31p automated hematology analyzer (Sysmex Corporation, Kobe, Japan). The EDTA venous blood samples were processed directly by the analyzer within 4 h after venipuncture. Briefly, 60 µL of the venous blood sample was used for analysis by the XN-31p in Low Malaria (LM) mode. The EDTA capillary sample (20 μL) was diluted with CELLPACK DCL reagent (120 μL) within 30 min after the finger prick and run by the analyzer immediately after dilution in Pre-Dilution (PD) mode.

The samples were aspirated and diluted automatically by the analyzer, and staining for nucleic acids was performed. Subsequently, infected red blood cells (iRBCs) and white blood cells (WBCs) were detected by a violet semiconductor 405 nm laser beam in the M channel. The data were automatically determined by analyzing the plotted scattergrams with forward-side scattered light and side fluorescent light, which allowed visualization of the iRBC population separate from the RBC population (Supplementary Fig. 2). The XN-31p output data separately report the following: 1) a complete blood count (CBC); 2) the presence of iRBCs: index test positive if “MI-RBC present” was displayed (iRBC ≥ LOQ; LoQ for LM mode: 20 iRBCs/μL, LoQ for PD mode: 40 iRBCs/μL), and index test negative or inconclusive otherwise (MI-RBC Abn Scattergram); 3) parasite count, both as a percentage of infected red blood cells (“MI-RBC%”) and absolute parasite density (“MI-RBC#”), expressed as %iRBC and iRBC/μL, respectively; and 4) Plasmodium species determined by flagging mechanisms using the M channel (applicable only for samples with MI-RBC# ≥ 100/μL): supplementally, it shows information on *P. falciparum* if the “Malaria?(P.f.)” flag is displayed, on other non-*P. falciparum* species if the “Malaria?(others)” flag is displayed, and on unclassified species if the “Malaria?(UNC)” flag is present. Mixed infections according to the index test were considered when both *P. falciparum* and other non-*P. falciparum* species were flagged simultaneously in a sample. All the results were automatically downloaded from the instrument as an Excel file that was exported and sent weekly to the data manager at the University of Antioquia.

#### Microscopy

Two thick blood films and one thin film were prepared from capillary samples from all participants and stained with Giemsa according to international guidelines^19^. A malaria microscopist read the thick- and thin-film slides at the field site to establish the malaria diagnosis. Later, a second expert malaria microscopist read all the slides at the reference laboratory in a blind manner. A third blind reader resolved any discrepancies (positive vs negative and differences in detected species). The microscopist in the field had 2 years of demonstrated expertise in malaria diagnosis and the two microscopists at the reference laboratory had 8 years and were classified as level expertise 1 according to WHO^19^.

A stained, thick blood smear was used to establish positive and negative judgment; the smear was considered negative if, after completing examination of 500 fields at 100 × magnification, no parasites (asexual or sexual) were found. A second thick smear was used as a backup for cases where there was any problem with staining or doubts with judgment. Mixed infection was determined according to WHO criteria^20^. For positive slides, the stained thin smear was used to confirm species diagnosis, and parasite density was calculated from both thick and thin films as previously described^19^. The thick films were used to count the number of asexual and sexual parasites per 500 leukocytes (when count was > 500 parasites/µL) or 1,500 leukocytes (when count was < 500 parasites/µL); parasite density was estimated using WBC count obtained by the XN-450 for each participant. The parasitemia from the thin film was calculated by establishing the percentage of infected red blood cells (iRBCs) among 10,000 counted erythrocytes. The parasitemia obtained by the second microscopist was used for comparison with the iRBC% and iRBC# calculated by the XN-31p.

#### Rapid diagnostic test (RDT)

Rapid diagnostic tests (SD BIOLINE Pf/Pv (HRP2/LDH), Ref 05KF80) were performed on EDTA-anticoagulated venous blood within 30 min after venipuncture, according to recommendations from the manufacturer. The RDTs were performed simultaneously with the sample processing by the Sysmex analyzers and before the MI. The test line reactivity of *Plasmodium falciparum* histidine-rich protein-2 (HRP2) and *Plasmodium vivax* parasite-lactate dehydrogenase (LDH) was scored. Any visible test line was considered positive, and the results were reported as *P. falciparum, P. vivax*, mixed infection (Pf/Pv) or negative.

#### Real-time polymerase chain reaction (qPCR)

Plasmodium genus detection: Molecular diagnosis was carried out in the reference laboratory in a blinded manner. DNA was extracted from a 160 µL aliquot of EDTA-venous blood after defrosting using a Qiagen QIAamp DNA Mini Kit (Catalog no 51106). An aliquot of 5 µL of DNA was used to amplify a region of the multicopy 18S rRNA gene from the genus Plasmodium by qPCR, as described by Rougemont et al., using Platinum Quantitative PCR superMix-UDG TaqMan Universal Master Mix (Invitrogen Thermo Scientific), primers and probes as previously reported^21^. Reactions were run on an Agilent AriaMx Real-Time PCR System. A positive result was determined for samples with a cycle threshold (Ct) value > 35.1, which corresponds to a parasitemia of 0.5 parasites/µL (limit of quantification (LoQ)). A negative result was reported when the Ct value > 39.5. Samples with Ct values ≥ 35.1 and ≤ 39.5 were classified with a final diagnosis of inconclusive; DNA was detected, but it was under the LoQ (very low parasitemia); qPCR genus detection was subsequently repeated for these samples to confirm the final classification. Qualitative results were reported since quantitative results do not represent the count of iRBCs.

Plasmodium species detection: All samples with a final diagnosis of positive and inconclusive were processed with *P. falciparum* and *P. vivax* species amplification protocols using two independent qPCR assays with primers described by Sing et al.^22^ and 5 × Hot FIREPol EvaGreen qPCR Supermix. Only samples that had not been amplified by *P. falciparum* or *P. vivax* protocols were additionally processed by qPCR for *P. malariae* using primers described by Sing et al.^22^ and detected by EvaGreen. If the Plasmodium species result persisted as inconclusive, nested PCR (nPCR) was carried out for *P. ovale* detection using a protocol reported by Sing et al.^22^. A positive result for *P. falciparum, P. vivax* or *P. malariae* species was defined according to the Ct, last relative fluorescence unit (ΔR last) and temperature melting (Tm) values, and a positive result for *P. ovale* was defined by the presence of bands on the agarose gel. Mixed infection was determined by the presence of two or more positive results from independent species amplification protocols. Samples with positive and inconclusive results from the genus protocol but negative results from the four species protocols were classified as *Plasmodium spp*.

### Hematological parameters

The EDTA venous blood samples (25 μL) were processed by the automated hematology analyzer XN-450 (Sysmex Corporation, Kobe, Japan) in WB mode. The samples were processed within 4 h after venipuncture, with a maximum interval of 30 min for processing between the XN-31p and XN-450. The data for the parameters white blood cell count (WBC), red blood cell count (RBC), hemoglobin (Hb), hematocrit and platelet count (PLT) were collected from the XN-450 analyzer to describe the study participants. Anemia was defined as Hb < 11 g/dL, thrombocytopenia as PLT < 150*10^3^/μL and severe thrombocytopenia as PLT < 50*10^3^/μL^23,24^.

### Data management and statistical analysis

Demographic, clinical, and epidemiological information was registered in a Microsoft Access 2010 database in the field. The database was sent weekly to the data manager at the University of Antioquia, who completed it with reference laboratory results (MI and qPCR) as well as results from the XN-31p.

Descriptive analysis was carried out for the participant characteristics. Independent analysis of the diagnostic accuracy of the XN-31p was performed for genus Plasmodium, *P. falciparum* and *P. vivax* species. For the last item, detection was assumed when the flagging mechanism for non-*P. falciparum* species was positive.

For the performance of the XN-31p (index test) with venous blood samples, sensitivity (SE), specificity (SP), positive/negative predictive values (PPV, NPV) and likelihood ratio of positive and negative tests (LRP and LRN) were calculated in reference to qPCR (gold standard test). In addition, the performance of the XN-31p was compared with that of MI and RDT because these are the currently available tests in the field. The McNemar test was applied to compare the SE and SP of the XN-31p, MI and RDT, all in reference to qPCR. Inconclusive results from genus qPCR were considered positive since these samples had Plasmodium DNA at very low concentrations (sometimes the result was not reproducible), and the strictest result was assumed to evaluate the performance of the analyzer. Independent analysis for genus detection was carried out assuming three scenarios for the MI-RBC Abn Scattergrams (index test inconclusive): positive, negative or excluded from analysis. The performance of the XN-31p for *P. falciparum* and *P. vivax* detection was assessed after excluding mixed infections from the results of the reference method. The intraclass correlation coefficient (ICC)^25^ and Bland–Altman plot^26^ were used to evaluate the concordance in the parasitemia between the XN-31p and MI among positive samples for both methods. The 95% confidence interval (95% CI) was estimated for each parameter.

For the performance of the XN-31p with finger-prick blood samples, SE, SP, PPV, NPV, LRP and LRN and their 95% CIs were calculated in reference to the qPCR results. Concordance in the malaria diagnosis and parasitemia between venous blood and finger-prick samples was evaluated by Cohen's kappa coefficient and the ICC/Bland–Altman plot method, respectively.

Descriptive analysis was carried out using IBM SPSS statistics program version 24 licensed by University of Antioquia, and performance analysis of the diagnostic tests was carried out using R version 3.6.3 and RStudio cloud programs with the following packages: epiR version 1.0–14^27^ for calculating SE, SP, PPV, NPV, LRP and LRN; DTComPair version 1.0.3^28^ for comparing SE and SP with the McNemar test; blandr^29^ for generating the Bland–Altman plot; irr^30^ for calculating the intraclass coefficient; and psych^31^ for calculating Cohen's kappa coefficient.

### Ethics approval and consent to participate

The study was reviewed and approved by the Facultad de Medicina Ethics Committee at the Universidad de Antioquia, Medellín, Colombia (Record 007; 11th May 2017), and Sysmex Corporation received approval from the Ethics Review Committee of the Japanese Association for the Promotion of State of the Art in Medicine (Approval date: 17th May 2017). This study complied with the principles stated in the Declaration of Helsinki (Ethical Principles for Medical Research Involving Human Subjects), the Belmont report (respect for persons, beneficence, and justice) and resolution 8430/1993 of the Ministry of National Health of Colombia (Chapter I: Of the ethical aspects of research in human beings). Before starting any study procedure, written informed consent was obtained from all participants. For participants < 18 years old, additional informed consent from their parents or legal guardian was also obtained. All the methods were carried out in accordance with the approved guidelines. Recorded data from participants were anonymized using a code. Participants were diagnosed by microscopy and treated according to the malaria guidelines from the Health Ministry of Colombia. The results from the XN-31p were not used to manage patients since this was the exact instrument under evaluation in this study. The information obtained from the questionnaires and clinical evaluations was stored and guarded in the Malaria Group of the University of Antioquia in Medellín.

## Results

### Description of participants and malaria frequency

A total of 1,967 eligible participants were enrolled, and 1754 blood samples were analyzed by the XN-31p. No samples were excluded from analysis due to inadequate sample storage, sample mismatch, hemolysis or a shortage of sampled blood. Only one sample had missing MI data.

Baseline demographic and clinical characteristics and data on malaria history are presented in Table 1 according to inclusion or exclusion based on the results of the index test (XN-31p). Although the goal of this study was not to address the clinical aspects of the malaria patients, the data show that the majority had signs and symptoms of uncomplicated malaria. Based on the clinical evaluation, hemoglobin levels and platelet counts, the frequency of warning signs or severe malaria was less than 2.0%.

**Table 1:** Baseline demographic and clinical characteristics of the enrolled febrile participants.

	Included in the XN-31p analysis (n = 1754)	Excluded from the XN-31p analysis (n = 213)	Total enrolled (n = 1967)
Age; median (IQR)	27 (14–44)	26 (14.5–41)	27 (14–43)
Male; n (%)	895 (51.03)	113 (53.1)	1008 (51.2)
Previous days with fever; median (IQR)	3 (3–5)	4 (3–5)	3 (3–5)
**Fever pattern, n (%)**			
Daily	1558 (88.8)	190 (89.2)	1748 (88.8)
Every two days	64 (3.6)	4 (1.9)	68 (3.5)
Irregular	132 (7.5)	19 (8.9)	151 (7.7)
Afro-Colombian; n (%)	1572 (89.6)	177 (83.1)	1749 (88.9)
Pregnant; n (%)	16 (0.91)	1 (0.5)	17 (0.9)
**Main occupation; n (%)**			
Farmer	39 (2.2)	5 (2.3)	44 (2.2)
Miner	182 (10.4)	19 (8.9)	201 (10.2)
Housewife	366 (20.9)	39 (18.3)	405 (20.6)
Student	532 (30.3)	65 (30.5)	597 (30.4)
Other	497 (28.3)	72 (33.8)	569 (28.9)
Unemployed	138 (7.9)	13 (6.1)	151 (7.7)
**Place of residence; n (%)**			
Quibdó	1654 (94.3)	201 (94.4)	1855 (94.3)
Other places inside Chocó	98 (5.6)	12 (5.6)	110 (5.6)
Outside Chocó	2 (0.1)	0 (0)	2 (0.05)
Rural area; n (%)	6 (0.34)	2 (0.9)	8 (0.1)
Residence time in years; median (IQR)	7 (2–16)	5 (2–14)	6 (2–16)
Travel to other endemic regions during last month; n (%)	507 (28.9)	59 (27.7)	566 (28.8)
Previous diagnosis of malaria (self-report); n (%)	1025 (58.4)	134 (62.9)	1159 (58.9)
Intake of antimalarials drugs over the last 4 weeks; n (%)	22 (1.3)	5 (2.3)	27 (1.3)
**Hematological parameters**^¥^			
White blood cell (10^3^/µL); median (IQR)	6.5 (4.91–8.74)	6.23 (4.92–7.73)	6.41 (4.91–8.61)
Red blood cell (10^6^/µL); median (IQR)	4.53 (4.19–4.93)	4.56 (4.2–5)	4.54 (4.18–4.93)
Hemoglobin (g/dL); median (IQR)	12.9 (11.7–14.1)	12.9 (11.8–14.3)	12.9 (11.7–14.2)
Anemia (Hb < 11 g/dL); n (%)	232 (13.2)	35 (16.4)	267 (13.6)
Hematocrit (%); median (IQR)	36.8 (33.6–40.1)	36.9 (33.7–40.8)	36.8 (33.6–40.2)
Platelets (10^3/µL); median (IQR)	231 (169–291)	208 (149–262.5)	229 (168–289)
Thrombocytopenia (< 150*10^3^/µL); n (%)	330 (18.8)	54 (25.4)	384 (19.5)
Severe thrombocytopenia (< 50*10^3^/µL); n (%)	23 (1.3)	2 (0.9)	25 (1.3)

^¥^Hematological parameters were collected from an XN-450.

*IQR* Interquartile range.

The results of the malaria diagnosis test for the enrolled febrile participants are presented in Table 2. The percentage of samples diagnosed with malaria according to MI was 30.3% (531/1754), with parasite densities ranging from 11 to 198,736 (median 3536; IQR 928–9984). A total of 33% (579/1754) of participants had a positive qPCR result, and submicroscopic infection (positive qPCR with negative MI) was detected in 2.7% (48/1754) of participants. All MI-positive samples were detected by qPCR. The RDT was positive for 29.2% (513/1754) of participants, and the XN-31p reported 29.4% (515/1754) positive samples, with parasite densities ranging from 22 to 291,303 (median 3508; IQR 886–10,613).

**Table 2:** Frequency of malaria among the enrolled febrile participants (n = 1754).

	XN-31p	MI	RDT	qPCR
Plasmodium positive; n (%)	515 (29.4)^€^	531 (30.3)	513 (29.2)	579 (33)
*P. falciparum*	340 (69.1)	393 (74)	387 (75.4)	394 (68)
*P. vivax*	121 (24.6)^¥^	118 (22.2)	118 (23)	128 (22.1)
Mixed Pf/Pv	22 (4.5)	20 (3.8)	8 (1.6)	41 (7.1)
*P. malariae*	NA	0 (0)	NA	0 (0)
*P. ovale*	NA	0 (0)	NA	0 (0)
Other non-classified species	9 (1.8)	0 (0)	NA	16 (2.8)
Parasitemia (parasites/µL); median (IQR)	3508 (886–10613)	2415 (598–7121)		
Parasitemia (%iRBC); median (IQR)	0.081 (0.02–0.24)	0.07 (0.02–0.24)		

*IQR* Interquartile range.

^€^Plasmodium species were not identified in 23 samples that had < 100 iRBCs/μL: according to the flagging rule, malaria species are not identified when the number of iRBCs is less than 100/μL.

^¥^The XN-31 prototype shows the “Malaria?(others)” flag when non-*P. falciparum* species are suspected.

^£^Plasmodium DNA detected but at levels lower than the LoQ was reported for 39 samples (2.2%).

### Diagnostic accuracy of the XN-31 prototype for iRBC detection

**Figure 1:**
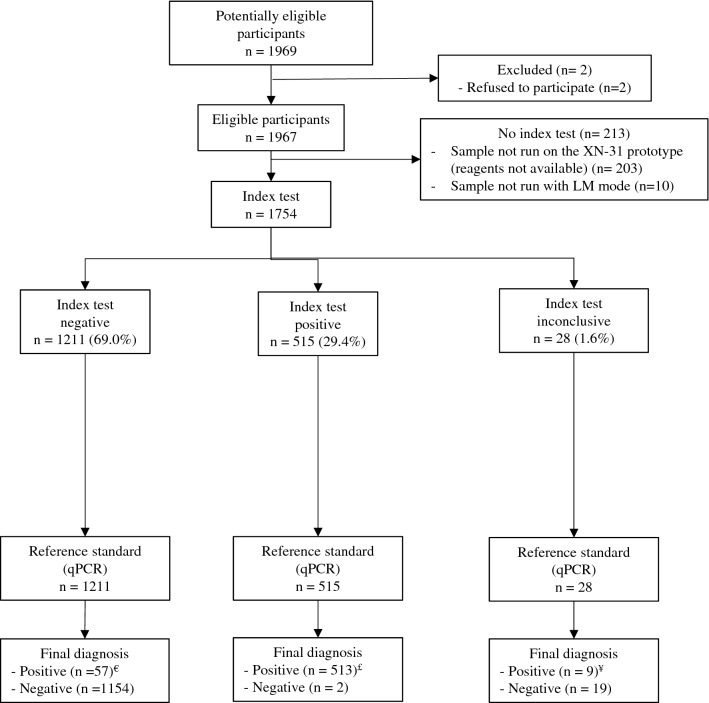
Flow chart of participants enrolled for the study of the diagnostic accuracy of the XN-31 prototype. ^€^36 samples with Ct levels lower than the LoQ but with DNA detected. ^£^2 samples with Ct levels lower than the LoQ but with DNA detected. ^¥^1 sample with Ct levels less than the LoQ but with DNA detected.

A total of 1,754 samples from febrile participants were included for the evaluation of the performance of the XN-31p with respect to the qPCR results in a clinical setting from a malaria-endemic region in Colombia (Fig. 1).

The percentage of inconclusive results from the XN-31p was 1.6% (28/1754); the demographic characteristics and history of malaria episodes of these participants were similar to those with valid results (positive or negative). However, some differences were observed among a number of hematological parameters (WBC, RBC, hematocrit and anemia) (Supplementary Table 1). Excluding those samples with inconclusive index tests, the percentage of false positive and false negative samples analyzed by the XN-31p was 0.12% (2/1726) and 3.3% (57/1726), respectively.

The parasite densities of the two false positive samples detected by the XN-31p were 30 and 40 parasites/µL. Here, the XN-31p detected iRBCs, but the flag mechanisms for detecting species were not indicated; in addition, both samples had high WBC counts (22.7*10^3^/µL and 25.8*10^3^/µL, respectively). On the other hand, 82.5% (47/57) of the false negative samples detected by the XN-31p had submicroscopic infections, and 76.6% (36/47) of them had a Ct level less than the LoQ from qPCR. The false-negative samples with microscopic infections (17.5%; 10/57) had parasite densities ranging from 16 to 261 parasites/µL; only three of these samples had parasitemia higher than the LoQ of the XN-31p (20 iRBCs/µL). In addition, the percentages by species among these samples were 57.9% (33/57) *P. falciparum*, 15.8% (9/57) *P. vivax*, 1.7% (1/57) mixed Pf/Pv and 24.6% (14/57) *Plasmodium spp*.

Table 3 presents the performance of the XN-31p in comparison with qPCR, MI and RDT for Plasmodium detection. The SE and SP of the XN-31p with respect to qPCR were 90.0% and 99.83%, respectively. The SE and SP of XN-31p with respect to MI were 98.09% and 99.83%, respectively, and similar results were found with respect to RDT.

**Table 3:** Performance of the XN-31 prototype with respect to MI, qPCR and RDT in detecting Plasmodium infection.

		MI		qPCR		RDT	
		POSITIVE	NEGATIVE	POSITIVE	NEGATIVE	POSITIVE	NEGATIVE
XN-31p*	POSITIVE	513	2	513	2	492	23
	NEGATIVE	10	1201	57	1154	14	1197
		**Value**	**95% CI**	**Value**	**95% CI**	**Value**	**95% CI**
	Sensitivity	98.09	(96.51–99.08)	90.00	(87.24–92.34)	97.23	(95.4–98.48)
	Specificity	99.83	(99.4–99.98)	99.83	(99.38–99.98)	98.11	(97.18–98.8)
	PPV	99.61	(98.6–99.95)	99.61	(98.6–99.95)	95.53	(93.37–97.15)
	NPV	99.17	(98.49–99.6)	95.29	(93.94–96.42)	98.84	(98.07–99.37)
	LRP	590	(147.72–2356.48)	520.2	(130.22–2078.056)	51.58	(34.4–77.33)
	LRN	0.02	(0.01–0.04)	0.1	(0.08–0.13)	0.03	(0.02–0.05)

*MI-RBC Abn Scattergrams were excluded.

Supplementary Table 2 shows the performance of MI and RDT with respect to qPCR, with SEs of, respectively, 91.71 and 87.39, and SPs of 100 and 99.4, which were similar to the SEs and SPs of the XN-31p. The performance results from the XN-31p were similar when samples with an inconclusive index test (MI-RBC Abn Scattergram) were considered positive or negative (data not shown).

### Diagnostic accuracy of the XN-31 prototype for Plasmodium species detection

The performance of the XN-31p with flagging information for the detection of Plasmodium species was evaluated for the detection of *P. falciparum* and *P. vivax*, the most common species that cause malaria in humans. Table 4 shows the performance of the XN-31p with respect to MI, qPCR and RDT.

**Table 4:** Performance of the XN-31 prototype in *Plasmodium* species detection.

		MI		qPCR		RDT	
		POSITIVE	NEGATIVE	POSITIVE	NEGATIVE	POSITIVE	NEGATIVE
***Plasmodium falciparum***^**€**^							
XN-31p	POSITIVE	350	5	330	6	347	12
	NEGATIVE	40	1312	61	1289	38	1322
		**Value**	**95% CI**	**Value**	**95% CI**	**Value**	**95% CI**
	Sensitivity	89.74	(86.3–92.57)	84.4	(80.42–87.85)	90.13	(86.7–92.92)
	Specificity	99.62	(99.12–99.88)	99.54	(98.99–99.83)	99.1	(98.43–99.53)
	PPV	98.59	(96.74–99.54)	98.21	(96.15–99.34)	96.66	(94.23–98.26)
	NPV	97.04	(95.99–97.88)	95.48	(94.23–96.53)	97.21	(96.18–98.02)
	LRP	236.38	(98.49–567.34)	182.16	(81.9–405.18)	100.19	(56.99–176.15)
	LRN	0.1	(0.08–0.14)	0.16	(0.12–0.2)	0.1	(0.07–0.13)
***Plasmodium vivax***^**€**^							
XN-31p	POSITIVE	112	17	114	17	111	27
	NEGATIVE	2	1576	10	1545	3	1578
		**Value**	**95% CI**	**Value**	**95% CI**	**Value**	**95% CI**
	Sensitivity	98.25	(93.81–99.79)	91.94	(85.67–96.06)	97.37	(92.5–99.45)
	Specificity	98.93	(98.3–99.38)	98.91	(98.26–99.36)	98.32	(97.56–98.89)
	PPV	86.82	(79.74–92.13)	87.02	(80.04–92.26)	80.43	(72.83–86.69)
	NPV	99.87	(99.54–99.98)	99.36	(98.82–99.69)	99.81	(99.45–99.96)
	LRP	92.06	(57.34–147.81)	84.47	(52.5–135.92)	57.88	(39.77–84.23)
	LRN	0.02	(0–0.07)	0.08	(0.05–0.15)	0.03	(0.01–0.08)

^€^Mixed infections according to the reference method were excluded for comparisons.

The SE and SP of the XN-31p with respect to qPCR in the detection of *P. falciparum* infections were 84.4 and 99.54, respectively. With respect to qPCR, the SE of MI and RDT for *P. falciparum* were higher than that of XN-31p (93.89% and 90.61%), while the SP was similar for the three methods (Supplementary Table 3). A total of 0.36% (6/1686) samples were falsely positive for *P. falciparum*, according to qPCR species detection, of which five had a *P. vivax* and one a *Plasmodium spp.* infection; all false positive samples were positive according to MI as well (four had a *P. vivax,* one had a *P. falciparum* and one had a mixed (Pf/Pv) infection). The false negative rate for *P. falciparum* was 3.6% (61/1,686); of these samples, 83.6% (51/61) had iRBCs with < 100/μl, and iRBCs were detected (> 20 iRBCs/µL) among only 45.9% (28/61).

The SE and SP of the XN-31p with respect to qPCR in the detection of *P. vivax* infections were 91.94 and 98.91, respectively. Both values were similar to those for MI (SE 92.31% and SP 100%), and the SE was better than that for RDT (85.94) in detecting *P. vivax* (Supplementary Table 3). A total of 1.01% (17/1,686) false positive samples were detected for *P. vivax*. According to qPCR species detection, all of them had a *P. falciparum* infection and were positive according to MI as well (16 had a *P. falciparum* and one a mixed Pf/Pv infection). The false negative rate for *P. vivax* was 0.59% (10/1686); all samples had iRBCs with < 100/μL, but iRBCs could not be detected among 90.0% (9/10) of the samples (< 20 iRBCs/µL).

### Concordance in the parasitemia quantification in venous blood between the XN-31 prototype and MI

**Figure 2:**
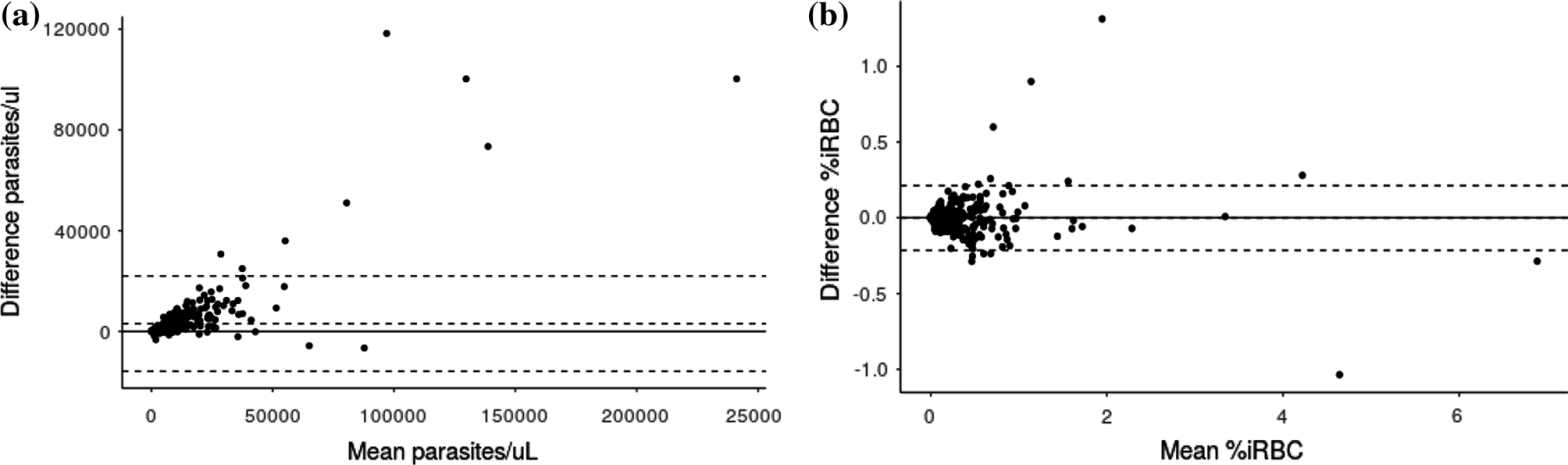
Bland–Altman plot for concordance in the parasitemia between the XN-31 prototype and microscopy*.* The figure shows the concordance in the parasitemia between the XN-31 prototype and microscopy from thick smears (parasites/µL) (n = 512) (**a**) and thin smears (iRBC%) (n = 513) (**b**), according to the Bland–Altman method. The difference in the parasitemia was calculated as (parasitemia according to the XN-31 prototype minus parasitemia according to MI).

The concordance in the parasitemia between the XN-31p (MI-RBC#/µL) and the thick blood smear was good, with an ICC of 0.85 and an average difference of − 3096 parasites/µL. In addition, there was excellent concordance in the parasitemia between the thin blood smear and the XN-31p (MI-RBC%), with an ICC 0.98 and an average difference of − 0.0013% iRBCs (Table 5 and Fig. 2). Only 1.6% (8/512) and 2.5% (13/513) of samples fell outside of the concordance interval for comparison between thick and thin blood smears, respectively. These samples presented higher values for parasitemia and and lower hemoglobin levels than those within the concordance interval (data not shown). When samples outside of the concordance interval were excluded, the concordance of parasitemia between the XN-31p (MI-RBC#/µL) and the thick smears improved (ICC 0.942, 95% CI 0.931–0.951; average difference 2,084 parasites/µL, SD 3277 parasites/µL). In contrast, the comparison with the thin blood smear was relatively unchanged (ICC 0.983, 95% CI 0.980–0.986; average difference − 0.004%, SD 0.055%).

**Table 5:** Concordance in the parasitemia between the XN-31 prototype and microscopy.

	XN-31 prototype vs thick smear (n = 512)		XN-31 prototype vs thin smear (n = 513)	
	Value	95% CI	Value	95% CI
ICC	0.852	(0.827–0.874)	0.977	(0.973–0.981)
Mean difference in parasitemia	3095.81	(2257.49–3934.13)	− 0.0013	(− 0.0107–0.0082)
SD difference in parasitemia	9655.32		0.1089	
Concordance inferior limit	− 15828.22	(− 17260.92)–(− 14395.52)	− 0.2148	(− 0.2309)–(− 0.1986)
Concordance superior limit	22019.84	(20587.14–23452.54)	0.2122	(0.1961–0.2283)

### Concordance in the quantification of the parasitemia by the XN-31 prototype between venous blood and finger prick samples

The results of the malaria diagnosis test for the capillary samples are presented in Table 6. Excluding samples with inconclusive index tests, the sensitivity and specificity of the XN-31p in Plasmodium detection from finger-prick samples with respect to qPCR were 90.91% (95% CI 75.67–98.08) and 100% (95% CI 86.28–100), respectively.

**Table 6:** Performance of the malaria diagnosis tests from the pilot study and of the XN-31 prototype using finger-prick samples.

	XN-31 prototype finger prick^€^	XN-31 prototype venous blood	Microscopy	qPCR
Plasmodium positive; n (%)	30 (51.7)	31 (51.7)	30 (50.0)	34 (56.7)
*P. falciparum*	16 (53.3)	14 (45.2)	14 (46.7)	16 (47.1)
*P. vivax*	12 (40.0)	13 (41.9)	15 (50.0)	16 (47.1)
Mixed Pf/Pv	0 (0.0)	1 (3.2)	1 (3.3)	2 (5.8)
Other species non classified	2 (6.7)	3^£^ (9.7)	0	0 (0.0)
Parasitemia (parasites/µL); median (IQR)	6728.5 (1158.7–30513.0)	4703.0 (789.0–28635.0)	5912.0 (1028.0–28868.0)	
Parasitemia (%iRBC); median (IQR)	0.161 (0.031–0.707)	0.123 (0.019–0.711)	0.19 (0.03–0.72)	

*IQR* Interquartile range.

^€^3.3% of samples (2/60) had inconclusive index tests (MI-RBC Abn Scattergram).

^£^Plasmodium species was not determined in these samples, as they had < 100 iRBCs/μL.

The concordance in the parasitemia between the XN-31p with finger-prick samples (PD mode) and with venous samples (LM mode) for Plasmodium detection was very good (kappa coefficient 0.97; 95% CI 0.90–1.00). On the other hand, the concordance in the parasitemia according to MI-RBC #/µL and MI-RBC% was excellent, with an ICC of 0.995 (95% CI 0.989–0.997), an average difference of 1,085.3 parasites/µL (SD 4,829.93 parasites/µL) an ICC of 0.957 (95% CI 0.913–0.980) and an average difference of − 0.05% (SD 0.15%), respectively (Supplementary Table 4 and Supplementary Fig. 3).

## Discussion

Although Colombia is classified as a low and unstable malaria transmission region^1,32,33^, the area of Quibdó, where this study was conducted, reported a high risk of malaria in 2018 and 2019, with annual parasitic indexes (APIs) of 34.1 and 49.4 per 1000, respectively^17,18^. This condition could have favored the high percentage of malaria among our febrile participants (approximately 30%) and makes this study similar to others in high-transmission areas^10,34^. This study evaluated the diagnostic accuracy of the XN-31p in a prospective cohort of acute febrile patients who consulted a physician for medical care and were tested for malaria. This was an advantageous scenario for testing the performance of the XN-31p, since there was a chance to enroll patients with a wide range of clinical presentations. However, the clinical spectrum of participants in this study was ultimately very homogenous and typical of those with uncomplicated malaria, reflecting the expected percentage of severe malaria in Colombia of approximately 2%^18^.

The XN-31p showed good performance with respect to qPCR in diagnosing malaria (Plasmodium genus), comparable to that MI and RDT, conventional methods used in the field. Using a previously defined cutoff of 20 iRBCs/μL^11^, the SE and SP were high (90.0% and 99.8%, respectively), and the false positive rate was 0.12%, less than the limit of the WHO criteria for recommending the procurement of an RDT procurement (< 10%)^35^. The XN-31p requires mains electricity and is not portable; therefore, it is not considered a point of care test. However, it could be very useful for malaria screening among acute febrile individuals who seek care at health centers in malaria endemic areas, offering a malaria diagnosis and automated blood count simultaneously.

A false negative rate of 3.3% was found for the XN-31p, which may not be of concern for febrile individuals informed about potential warning signs and the need for serial diagnostic tests if their symptoms persist. On the other hand, in settings of active surveillance in low-transmission areas where low parasitemia is common (< 100 parasites/µL)^36^, the XN-31p may not be the best option for diagnosis, as a higher false positive rate is expected.

Approximately 2% of samples tested by the XN-31p had inconclusive results (MI-RBC Abn Scattergram); therefore, they would require another test. Hematological alterations in these participants could contribute to the difficulty of the analyzer in accurately classifying these samples. Pillay et al. 2019 reported a MI-RBC Abn Scattergram flag from an XN-31p in LM mode in 36% of samples with hematological alterations (low hemoglobin levels, low platelet counts, hemoglobinopathies and elevated reticulocyte counts)^11^. It is necessary to evaluate the performance of the XN-31p for participants with severe diseases to confirm the possible MI-RBC Abn Scattergrams caused by hematological alterations.

Current study employed expert microscopists to read all slides (Level 1)^19^, explaining the excellent performance of MI with respect to qPCR, in contrast to other studies^37^. It is possible that the performance of the XN-31p could be different than that reported here if compared with MI performed in field conditions.

When the results were evaluated by species, the performance of the XN-31p was variable; while the detection of *P. vivax* was equivalent to that of expert MI and RDT, *P. falciparum* detection was possible but with a lower performance than MI and RDT. The critical point for discriminating Plasmodium species based on the flagging mechanisms in this study was the level of parasitemia. The XN-31p can discriminate species when the parasitemia is higher than 100 iRBCs/µL; in the current study, the parasitemia for *P. falciparum* infections were lower than those for *P. vivax* infections. On the other hand, some *P. vivax* stages are larger in size than *P. falciparum*, which also favors the detection of *P. vivax* by the XN-31p. Additional effort is necessary to study the performance of the XN-31p with samples with ultralow parasitemia, mainly with *P. falciparum* infections. Moreover, these flagging mechanisms have not yet been fully validated for differentiating every species by the manufacturer due to a lack of data on rare species such as *P. malarie*, *P. ovale* or *P. knowlesi*. It is important to differentiate these species to determine appropriate treatment; therefore, further evaluation including these less frequent species should be performed to improve the species flagging mechanism.

The performance of the XN-31p in this epidemiological setting in Colombia is better than that reported by Post et al. 2019 in Burkina Faso (SE 90.% vs 70.9%)^10^, which may be because the Post et al. study was carried out in a setting with a higher percentage of complicated malaria (approximately 35%), a predominance of *P. falciparum* infections (99%) and a higher reporting of antimalarial uptake in the past two weeks; all conditions offer disadvantages for diagnosing malaria with a hematological analyzer.

The concordance in the parasitemia between the XN-31p and MI was very good, showing an important strength of the former because MI requires a longer reading time for the accurate estimation of parasitemia. It is important to consider that thin smear parasitemia estimates can be closer to XN-31p values since both methods count complete erythrocytes.

On the other hand, the XN-31p had malaria detection capabilities equivalent to those of qPCR and MI, even with finger-prick blood. At present, the XN-31p is intended to be used with venous blood, and it is important to accumulate positive results such as those obtained here to expand its use with finger-prick blood as well.

### Limitations

The fact that there were virtually no severe malaria cases represented in our cohort constitutes a limitation of this study since the XN-31p results cannot be generalized to this group of patients. It was not possible to determine the performance of the XN-31p for mixed infections, as the percentage of coinfections was low in this study and the number of samples with this condition was insufficient to guarantee the accuracy of the results. The pilot study for evaluating the concordance in the malaria diagnosis between venous blood and finger-prick blood from the XN-31p included a limited number of samples, which were selected according to different levels of parasitemia; this could represent a potential source bias.

## Conclusions

The XN-31p analyzer offers a rapid and accurate alternative method for malaria diagnosis in clinical laboratories located in endemic areas, although an economic evaluation study should be carried out. This analyzer is comparable to expert MI in the detection of Plasmodium infections and the quantification of parasitemia; in addition, it performs a complete blood-cell count and produces a results report in one minute. The XN-31p is able to discriminate parasite species; however, it shows some room for improvement in detecting *P. falciparum*. The performance of the XN-31p in the discrimination of coinfections needs to be evaluated in future studies.

## Supplementary Information

Supplementary Information

## Data Availability

The datasets used and analyzed during the current study are available from the corresponding author on reasonable request.

## Abbreviations

CI: Confidence interval
Hb: Hemoglobin
IQR: Interquartile range
iRBC: Infected red blood cell
LoQ: Limit of quantification
LRP: Likelihood ratio of positive test
LRN: Likelihood ratio of negative test
MI: Microscopy
NPV: Negative predictive value
PPV: Positive predictive value
qPCR: Real-time polymerase chain reaction
RBC: Red blood cell
RDT: Rapid diagnostic test
SE: Sensitivity
SP: Specificity
WBC: White blood cell
