# Supplementary material for: Performance of the hematology analyzer XN-31 prototype in the detection of Plasmodium infections in an endemic region of Colombia
Source: Sci Rep. 2021 Mar 4;11:5268. doi: 10.1038/s41598-021-84594-y (PMC7933134; doi:10.1038/s41598-021-84594-y)
Supplement: Supplementary file 1 — Supplementary Information [file 41598_2021_84594_MOESM1_ESM.docx]

**Supplementary figures and tables**

Title: Performance of the hematology analyzer XN-31 prototype in the detection of Plasmodium infections in an endemic region of Colombia

Authors: Lina Zuluaga-Idárraga, Alexandra Rios, Verónica Sierra, Edwar Garzón, Alberto Tobón-Castaño, Ikki Takehara, Yuji Toya, Munehisa Izuka, Kinya Uchihashi, Tatiana Lopera-Mesa.

Supplementary figure 1. Map of study site

Participants were recruited in a first level hospital in the urban center of Quibdó municipality, Chocó department (Colombia, South America). Images from <https://commons.wikimedia.org/wiki/File:Colombia_-_Choc%C3%B3_-_Quibd%C3%B3.svg> and <https://commons.wikimedia.org/wiki/File:COL_orthographic_(San_Andr%C3%A9s_and_Providencia_special).svg>

Supplementary figure 2. Scattergram from positive and negative samples by XN-31 prototype

Supplementary figure 3. Bland-Altman plot for concordance in the parasitemia from XN-31 prototype with finger prick vs venous samples.

The figure shows the concordance in the parasitemia XN-31 prototype with finger prick sample (PD mode) vs venous sample (LM mode) (a) iRBC/µL) (b) %iRBC, according to the Bland-Altman method. The difference in the parasitemia was calculated as (parasitemia according to the finger prick sample minus parasitemia according to venous sample).

Supplementary table 1. Baseline demographic and clinical characteristics of participants with MI-RBC Abn Scattergram

|  | **MI-RBC Abn Scattergram**  **(n = 28)** | | |  | **Positive/Negative scattergram**  **(n = 1726)** | | |  | **P value^¥^** |
| --- | --- | --- | --- | --- | --- | --- | --- | --- | --- |
| Age; median (IQR) | 35 | (17.25 - 35) | |  | 27 | | (14 - 27) |  | 0.180 |
| Male; n(%) | 17 | (60.7) | |  | 878 | | (50.9) |  | 0.301 |
| Previous days with fever; median (IQR) | 4.5 | (3 - 4.5) | |  | 3 | | (3 - 3) |  | 0.064 |
| Fever pattern, n(%) |  |  | |  |  | |  |  |  |
| Daily | 21 | (75) | |  | 1537 | | (89) |  | **0.007** |
| Every two days | 4 | (14.3) | |  | 60 | | (3.5) |  |  |
| Irregular | 3 | (10.7) | |  | 129 | | (7.5) |  |  |
| Afro-Colombians; n(%) | 24 | (85.7) | |  | 1548 | | (89.7) |  | 0.462 |
| Pregnant; n(%) | 1 | (3.6) | |  | 15 | | (0.9) |  | 0.228 |
| Main occupation; n(%) |  |  | |  |  | |  |  |  |
| Farmer | 1 | (3.6) | |  | 38 | | (2.2) |  | 0.904 |
| Miner | 2 | (7.1) | |  | 180 | | (10.4) |  |  |
| Housewife | 4 | (14.3) | |  | 362 | | (21) |  |  |
| Student | 9 | (32.1) | |  | 523 | | (30.3) |  |  |
| Other | 9 | (32.1) | |  | 488 | | (28.3) |  |  |
| Unemployed | 3 | (10.7) | |  | 135 | | (7.8) |  |  |
| Place of residence; n(%) |  |  | |  |  | |  |  |  |
| Quibdó | 26 | (92.9) | |  | 1628 | | (94.3) |  | 0.873 |
| Other places inside Chocó | 98 | (7.2) | |  | 92 | | (5.3) |  |  |
| Outside Chocó | 0 | (0) | |  | 6 | | (0.3) |  |  |
| Rural area; n(%) | 1 | (3.6) | |  | 5 | | (0.3) |  | **0.012** |
| Residence time in years; median (IQR) | 9 | (3.25 - 9) | |  | 7 | | (2) |  | 0.322 |
| Travel to other endemic regions during last month; n(%) | 9 | (31.2) | |  | 498 | | (28.9) |  | 0.703 |
| Previous diagnosis of malaria (self-report); n(%) | 20 | (71.4) | |  | 1005 | | (58.2) |  | 0.160 |
| Intake of antimalarials drugs over the last 4 weeks; n(%) | 1 | (3.6) | |  | 21 | | (1.2) |  | 0.300 |
|  |  |  | |  |  | |  |  |  |
| Hematological parameters |  |  | |  |  | |  |  |  |
| White blood cell (10^3/µL); median (IQR) | 8.6 | (6.44 - 8.6) | |  | 6.41 | | (4.9 - 6.41) |  | **0.001** |
| Red blood cell (10^6/µL); median (IQR) | 4.06 | (3.45 - 4.06) | |  | 4.53 | | (4.19 - 4.53) |  | **0.003** |
| Hemoglobin (g/dL); median (IQR) | 12.3 | (9.2 - 12.3) | |  | 12.9 | | (11.7 - 12.9) |  | 0.181 |
| Anemia (Hb<11 g/dL); n(%) | 10 | (35.7) | |  | 222 | | (12.9) |  | **0.002** |
| Hematocrit (%); median (IQR) | 33.25 | (26.05 - 33.25) | |  | 36.8 | | (33.6 - 36.8) |  | **0.015** |
| Platelets (10^3/uL); median (IQR) | 280.5 | (195.75 - 280.5) | |  | 231 | | (169 - 231) |  | 0.057 |
| Thrombocytopenia (<150*10^3/uL); n(%) | 5 | (17.9) | |  | 325 | | (18.8) |  | 0.894 |
| Severe thrombocytopenia (<50*10^3/uL); n(%) | 0 | (0) | |  | 23 | | (1.3) |  | 0.538 |
|  |  |  | |  |  | |  |  |  |
| Plasmodium positive by microscopy; n(%) | 8 | (28.6) | |  | 523 | | (30.3) |  | 0.843 |
| Plasmodium positive by qPCR; n(%) | 9 | (32.1) | |  | 570 | | (33.0) |  | 0.922 |
| Plasmodium positive by RDT; n(%) | 7 | (25.0) | |  | 506 | | (29.3) |  | 0.618 |
| Parasitemia by thick smear; median (IQR) (parasites/µL) | 1,870 | (182 – 39,731) |  | 2,333 | | (549 – 7,068) | |  | 0.993 |
| Parasitemia by thin smear; median (IQR) (%iRBC) | 0.08 | (0.00 - 1.55) | |  | 0.07 | | (0.02 - 0.24) |  | 0.911 |

^¥^ Chi-squared test for qualitative variables and Mann-Whitney test for quantitative variables.

Supplementary table 2. Performance of the XN-31 prototype, MI and RDT with respect to qPCR in detecting Plasmodium infection.

|  | **XN-31p**^*^ | |  | **MI** | |  | **RDT** | |  | **McNemar test XN31p vs MI** | |  | **McNemar test XN31p vs RDT** | |
| --- | --- | --- | --- | --- | --- | --- | --- | --- | --- | --- | --- | --- | --- | --- |
|  | POSITIVE | NEGATIVE |  | POSITIVE | NEGATIVE |  | POSITIVE | NEGATIVE |  |  |  |  |  |  |
| POSITIVE | 513 | 2 |  | 531 | 0 |  | 506 | 7 |  |  |  |  |  |  |
| NEGATIVE | 57 | 1,154 |  | 48 | 1,175 |  | 73 | 1,168 |  |  |  |  |  |  |
|  |  |  |  |  |  |  |  |  |  |  |  |  |  |  |
|  | **Value** | **95% CI** |  |  |  |  |  |  |  | Difference | P value |  | Difference | P value |
| Sensitivity | 90.00 | (87.24 - 92.34) |  | 91.71 | (89.16 - 93.82) |  | 87.39 | (84.41 - 89.99) |  | 1.75 | 0.00157 |  | 2.46 | 0.00815 |
| Specificity | 99.83 | (99.38 - 99.98) |  | 100 | (99.69 - 100) |  | 99.4 | (98.78 - 99.76) |  | 0.17 | 0.157 |  | 0.43 | 0.0956 |
| PPV | 99.61 | (98.6 - 99.95) |  | 100 | (99.31 - 100) |  | 98.64 | (97.21 - 99.45) |  |  |  |  |  |  |
| NPV | 95.29 | (93.94 - 96.42) |  | 96.08 | (94.83 - 97.09) |  | 94.12 | (92.66 - 95.36) |  |  |  |  |  |  |
| LRP | 520.2 | (130.22 – 2,078.056) |  | - |  |  | 146.69 | (70.04 - 307.23) |  |  |  |  |  |  |
| LRN | 0.1 | (0.08 - 0.13) |  | 0.08 | (0.06 - 0.11) |  | 0.13 | (0.1 - 0.16) |  |  |  |  |  |  |

^*^ MI-RBC Abn Scattergram were excluded

Supplementary table 3. Performance of the XN-31 prototype, MI and RDT with respect to qPCR for Plasmodium species detection.

|  | **XN-31p** | |  | **MI** | |  | **RDT** | |  | **McNemar test XN31p vs MI** | |  | **McNemar test XN31p vs RDT** | |
| --- | --- | --- | --- | --- | --- | --- | --- | --- | --- | --- | --- | --- | --- | --- |
| ***Plasmodium falciparum*** | | | | | | | | | | | | | | |
|  | POSITIVE | NEGATIVE |  | POSITIVE | NEGATIVE |  | POSITIVE | NEGATIVE |  | **Difference** | **P value** |  | **Difference** | **P value** |
| POSITIVE | 330 | 61 |  | 369 | 24 |  | 357 | 37 |  |  |  |  |  |  |
| NEGATIVE | 6 | 1,289 |  | 1 | 1,307 |  | 8 | 1,311 |  |  |  |  |  |  |
|  | **Value** | **95% CI** |  |  |  |  |  |  |  |  |  |  |  |  |
| Sensitivity | 84.4 | (80.42 - 87.85) |  | 93.89 | (91.05 - 96.05) |  | 90.61 | (87.29 - 93.3) |  | 9.49 | < 0.0001 |  | 6.39 | < 0.0001 |
| Specificity | 99.54 | (98.99 - 99.83) |  | 99.92 | (99.57 - 100) |  | 99.39 | (98.81 - 99.74) |  | 0.031 | 0.0455 |  | 0.15 | 0.564 |
| PPV | 98.21 | (96.15 - 99.34) |  | 99.73 | (98.5 - 99.99) |  | 97.81 | (95.73 - 99.05) |  |  |  |  |  |  |
| NPV | 95.48 | (94.23 - 96.53) |  | 98.2 | (97.33 - 98.84) |  | 97.26 | (96.24 - 98.06) |  |  |  |  |  |  |
| LRP | 182.16 | (81.9 - 405.18) |  | 1,228.12 | (173.1 - 8,713.41) |  | 149.39 | (74.81 - 298.32) |  |  |  |  |  |  |
| LRN | 0.16 | (0.12 - 0.2) |  | 0.06 | (0.04 - 0.09) |  | 0.09 | (0.07 - 0.13) |  |  |  |  |  |  |
|  |  |  |  |  |  |  |  |  |  |  |  |  |  |  |
| ***Plasmodium vivax*** | | | | | | | | | | | | | | |
|  | POSITIVE | NEGATIVE |  | POSITIVE | NEGATIVE |  | POSITIVE | NEGATIVE |  |  |  |  |  |  |
| POSITIVE | 114 | 10 |  | 108 | 9 |  | 110 | 18 |  |  |  |  |  |  |
| NEGATIVE | 17 | 1,545 |  | 0 | 1,584 |  | 1 | 1,584 |  |  |  |  |  |  |
|  | **Value** | **95% CI** |  |  |  |  |  |  |  |  |  |  |  |  |
| Sensitivity | 91.94 | (85.67 - 96.06) |  | 92.31 | (85.9 - 96.42) |  | 85.94 | (78.69 - 91.45) |  | 0.87 | 0.317 |  | 6.45 | 0.0047 |
| Specificity | 98.91 | (98.26 - 99.36) |  | 100 | (99.77 - 100) |  | 99.94 | (99.65 - 100) |  | 1.02 | < 0.0001 |  | 1.02 | 0.0002 |
| PPV | 87.02 | (80.04 - 92.26) |  | 100 | (96.64 - 100) |  | 99.1 | (95.08 - 99.98) |  |  |  |  |  |  |
| NPV | 99.36 | (98.82 - 99.69) |  | 99.44 | (98.93 - 99.74) |  | 98.88 | (98.23 - 99.33) |  |  |  |  |  |  |
| LRP | 84.47 | (52.5 - 135.92) |  | - |  |  | 1,362.11 | (191.75 - 9,675.85) |  |  |  |  |  |  |
| LRN | 0.08 | (0.05 - 0.15) |  | 0.08 | (0.04 - 0.14) |  | 0.14 | (0.09 - 0.22) |  |  |  |  |  |  |

Supplementary table 4. Concordance of parasitemia between XN-31 prototype with finger prick sample and venous sample.

|  | **Parasitemia by MI-RBC # /µL** | |  | **Parasitemia by iRBC%** | |
| --- | --- | --- | --- | --- | --- |
|  | **Value** | **95% CI** |  | **Value** | **95% CI** |
| ICC | 0.995 | (0.989 - 0.997) |  | 0.957 | (0.913 - 0.980) |
| Mean difference of parasitemia | 1085.3 | (-718.23 - 2888.83) |  | -0,05 | (-0.16 - 0.06) |
| SD difference of parasitemia | 4829.93 |  |  | 0.30 |  |
| Concordance inferior limit | -8381,18 | (-11463.43 - -5298.93) |  | -0.64 | (-0.83 - -0,45) |
| Concordance superior limit | 10551.78 | (7469.53 - 13634.03) |  | 0.54 | (0.35 - 0.73) |
